# Category-Aware Two-Stage Divide-and-Ensemble Framework for Sperm Morphology Classification

**DOI:** 10.3390/diagnostics15172234

**Published:** 2025-09-03

**Authors:** Aydın Kağan Turkoglu, Gorkem Serbes, Hakkı Uzun, Abdulsamet Aktas, Merve Huner Yigit, Hamza Osman Ilhan

**Affiliations:** 1 Department of Computer Engineering, Faculty of Electrical and Electronics, Yildiz Technical University, 34220 Istanbul, Turkey; kagan.turkoglu@std.yildiz.edu.tr (A.K.T.); hoilhan@yildiz.edu.tr (H.O.I.); 2 Department of Biomedical Engineering, Faculty of Electrical and Electronics, Yildiz Technical University, 34220 Istanbul, Turkey; gserbes@yildiz.edu.tr; 3 Department of Urology, Faculty of Medicine, Recep Tayyip Erdoğan University, 53000 Rize, Turkey; 4 Department of Computer Engineering, Faculty of Technology, Marmara University, 34840 Istanbul, Turkey; abdulsamet.aktas@marmara.edu.tr; 5 Department of Medical Biochemistry, Faculty of Medicine, Recep Tayyip Erdoğan University, 53000 Rize, Turkey; merve.huner@erdogan.edu.tr

**Keywords:** sperm morphology, deep learning, two-stage classification, ensemble learning, vision transformers, NFNet

## Abstract

**Introduction:** Sperm morphology is a fundamental parameter in the evaluation of male infertility, offering critical insights into reproductive health. However, traditional manual assessments under microscopy are limited by operator dependency and subjective interpretation caused by biological variation. To overcome these limitations, there is a need for accurate and fully automated classification systems. **Objectives:** This study aims to develop a two-stage, fully automated sperm morphology classification framework that can accurately identify a wide spectrum of abnormalities. The framework is designed to reduce subjectivity, minimize misclassification between visually similar categories, and provide more reliable diagnostic support in reproductive healthcare. **Methods:** A novel two-stage deep learning-based framework is proposed utilizing images from three staining-specific versions of a comprehensive 18-class dataset. In the first stage, sperm images are categorized into two principal groups: (1) head and neck region abnormalities, and (2) normal morphology together with tail-related abnormalities. In the second stage, a customized ensemble model—integrating four distinct deep learning architectures, including DeepMind’s NFNet-F4 and vision transformer (ViT) variants—is employed for detailed abnormality classification. Unlike conventional majority voting, a structured multi-stage voting strategy is introduced to enhance decision reliability. **Results:** The proposed framework consistently outperforms single-model baselines, achieving accuracies of 69.43%, 71.34%, and 68.41% across the three staining protocols. These results correspond to a statistically significant 4.38% improvement over prior approaches in the literature. Moreover, the two-stage system substantially reduces misclassification among visually similar categories, demonstrating enhanced ability to detect subtle morphological variations. **Conclusions:** The proposed two-stage, ensemble-based framework provides a robust and accurate solution for automated sperm morphology classification. By combining hierarchical classification with structured decision fusion, the method advances beyond traditional and single-model approaches, offering a reliable and scalable tool for clinical decision-making in male fertility assessment.

## 1. Introduction

Male infertility is a major global health concern, affecting approximately 17.5% of adults and making semen analysis—particularly sperm morphology evaluation—a critical diagnostic tool [1,2]. Manual microscopic assessment is often subjective and inconsistent, while computer-aided sperm analysis (CASA) systems, though widely used, are costly, inflexible, and limited in functionality, particularly when analyzing noisy or low-quality samples [3,4]. Furthermore, most CASA systems focus primarily on assessing motility and vitality in fresh, unstained semen, overlooking subtle morphological details. In contrast, the World Health Organization (WHO) recommends using stained and fixed smears, as staining reveals fine morphological defects that are otherwise difficult to detect [1].

Recent deep learning approaches (convolutional neural networks and vision transformers) have improved sperm morphology classification performance but still face challenges due to class imbalance and high inter-class similarity limitations of existing open-source datasets [5,6,7,8,9,10,11,12]. To be able to overcome the limitations of existing sperm morphology analysis datasets, the Hi-LabSpermMorpho dataset, providing a large-scale, expert-labeled dataset with 18 distinct sperm morphology classes, was introduced [2].

Inspired by hierarchical “splitter” models [13,14,15], we adopt a two-stage divide and ensemble framework: a splitter first routes images to Category 1 (head/neck abnormalities) or Category 2 (tail abnormalities and normal sperm), and category-specific ensembles perform fine-grained classification. This approach delivers a 4–5% accuracy improvement over single-model baselines without requiring additional data.

The key contributions of this study are as follows:

- A two-stage classification framework is proposed that first classifies samples into two major categories before detailed classification, improving model robustness and prediction accuracy.
- A multi-staged ensemble voting mechanism is introduced to increase classification reliability by allowing models to use both primary and secondary votes.
- Multiple deep learning architectures are evaluated, with NFNet-based models identified as particularly effective for sperm morphology classification.
- The benefits of ensemble learning are demonstrated, showing that using an even-numbered ensemble with a structured voting strategy leads to superior performance compared to traditional odd-numbered ensembles.

Experimental results show that the proposed approach achieves higher classification accuracy compared to conventional single-model classifiers and unstructured ensembles. By addressing the challenges of sperm morphology classification, this study contributes to the development of more accurate and reliable deep learning-based sperm analysis.

Automated sperm morphology classification has become an active research area due to its clinical relevance. Recent studies have utilized machine learning and deep learning methods to reduce subjectivity and manual effort in morphology assessment. However, reliable classification of sperm abnormalities remains challenging due to the lack of consistent datasets and variability in staining techniques [2]. The fair comparison of reported accuracy values is obstructed by substantial differences in image types, dataset sizes, and class numbers across studies. Therefore, rather than comparing the accuracy metrics from different studies, this work focuses on investigating the methodologies employed in sperm morphology analysis.

Spencer et al. employed an ensemble CNN approach combining VGG, ResNet, and DenseNet architectures, along with a meta-classifier to improve accuracy over individual models [16]. Javadi et al. introduced a two-phase system that detects sperm head regions first and then classifies abnormalities, demonstrating that targeted region detection enhances classification accuracy, particularly for non-stained images [6].

Iqbal et al. developed a custom CNN optimized specifically for sperm head abnormalities, emphasizing computational efficiency and adherence to WHO criteria. They successfully addressed inter-class similarity and intra-class variability challenges, particularly with amorphous head types [17]. Similarly, Riordon et al. utilized transfer learning with the VGG16 model and saliency mapping to visually interpret model predictions and address limited dataset sizes through augmentation techniques [18].

Ilhan and Serbes proposed a different approach for sperm morphology analysis using deep learning. They applied a two-stage fine-tuning method in which two deep neural networks, VGG16 and GoogleNet, were first fine-tuned on a larger dataset named SMIDS, then fine-tuned again on smaller datasets like HuSHeM and SCIAN-Morpho. Their method also used an ensemble strategy, specifically soft voting, to combine predictions from both networks. Their methodology distinguishes itself by avoiding manual preprocessing, such as image rotation or cropping, thereby ensuring full automation and practicality in real-world applications. Experiments showed that using an extra dataset for fine-tuning and combining different model output significantly improved classification results, especially on datasets with fewer samples or low-quality images [19]. Our research and the work by Ilhan and Serbes [19] both employ ensemble learning to enhance sperm morphology classification. While their approach uses soft voting to aggregate predictions from multiple models, our method integrates majority voting within a two-stage classification pipeline. Both techniques aim to improve classification robustness with multiple models working together instead of a single architecture.

Chandra et al. explored the use of various deep learning frameworks to analyze sperm morphology, with the aim of automating the assessment of male infertility. They tested multiple well-known deep neural networks, such as VGG [20], ResNet [21], MobileNet [22], and DenseNet [23], in the classification of the MHSMA dataset [6]. A key aspect of their approach was the use of feature activation visualization to analyze sperm cell morphology and better understand model decisions, particularly focusing on how different structures affect performance when classifying sperm head, vacuole, and acrosome abnormalities. They visualized model predictions using Grad-CAM, which shows how neural networks interpret sperm images. In addition, they addressed common challenges such as unbalanced datasets and limited training samples by employing various data augmentation techniques. Their findings support the use of CNNs in sperm morphology classification and suggest that deep learning models can be used as a reliable tool for automated sperm analysis in both speed and consistency [24].

Hierarchical and divide-and-conquer methods have also been shown effective outside sperm morphology. Romero et al. applied a top-down hierarchical classification method to ensure consistency in gene function prediction, highlighting structured approaches for complex tasks [25]. Asafuddoula et al. proposed a divide-and-conquer ensemble classifier to specifically address class imbalance by optimizing individual class accuracy rather than overall accuracy [26]. Both studies support the idea that hierarchical and staged classification strategies improve performance for complex classification tasks.

To address these challenges mentioned in other studies, a hierarchical two-stage framework for sperm morphology classification is implemented in this study. The first stage utilizes a dedicated splitter model to broadly categorize sperm morphology, followed by an ensemble of four deep learning models per category in the second stage. Additionally, a multi-staged ensemble voting mechanism is introduced, where models cast primary and secondary votes to determine the final prediction. This voting strategy mitigates the influence of dominant classes and ensures more balanced decision-making across different sperm abnormalities. By using category-specific ensembles, a splitter model, and an optimized voting mechanism, a more structured and effective approach to the classification of sperm morphology is provided.

## 2. Materials and Methods

### 2.1. Dataset and Preprocessing

The dataset used in this study is the Hi-LabSpermMorpho dataset, which consists of expert-labeled RGB images of sperm morphology. According to the WHO Laboratory Manual [27], sperm morphology is evaluated based on defects in three anatomical regions: the head, midpiece (neck), and tail. Abnormal spermatozoa are classified according to malformations in these regions. Among these, head defects are by far the most frequently observed abnormalities. It has been reported that 85–92% of spermatozoa in teratozoospermic samples exhibit head defects. Specifically, amorphous (irregular-shaped) heads are the most common subtype, representing up to one-third of all head anomalies in some studies. Other prevalent head defects include tapered heads, pyriform heads, vacuolated heads, and double heads, all of which are linked to impaired fertilization potential. Midpiece (neck) anomalies are the second most frequent category. These include angular deviations, abnormally thin or thick necks, and persistent cytoplasmic droplets. The WHO recommends paying special attention to large cytoplasmic remnants, as they may reflect incomplete spermiogenesis and be associated with oxidative stress. Tail defects, while less frequent overall, are clinically significant due to their direct impact on motility. Common tail abnormalities include curly tails, short tails, duplicated tails, and broken or sharply bent tails, all of which hinder progressive motility. Similarly, in our study, the morphological classes used (normal, head abnormality, midpiece abnormality, tail abnormality) are fully consistent with the WHO 2021 classification. This subdivision is not only widely used in the literature but also enables clinically meaningful interpretation, as different types of defects can have distinct etiologies and implications for fertility.

The dataset is classified into 18 categories, depicting sperm head, neck, and tail abnormalities. These images were acquired using bright-field microscopy with a customized imaging setup incorporating a mobile phone camera. Three different Diff-Quick staining techniques (BesLab, Histoplus, and GBL) were used to enhance morphological features for classification [2].

Table 1 presents the class distribution of sperm morphology images across three different staining methods used in the Hi-LabSpermMorpho dataset: BesLab, Histoplus, and GBL. Each column corresponds to one staining technique, detailing the number of images available for each sperm morphology class, including various head defects (e.g., amorphous, tapered, vacuolated heads), neck abnormalities (e.g., twisted, thick, thin necks), tail defects (e.g., twisted, curly, short tails), as well as normal sperm morphology. The total number of images per staining method is also provided. The dataset is highly imbalanced: some classes (e.g., amorphous head and tapered head) contain thousands of samples, whereas others (e.g., thin neck and long tail) are significantly under-represented. This class imbalance poses challenges for deep learning models, potentially leading to biases in classification performance. Nevertheless, the comprehensive nature and diversity of the dataset across different staining protocols support the robustness of our classification approach.

**Figure 1:**
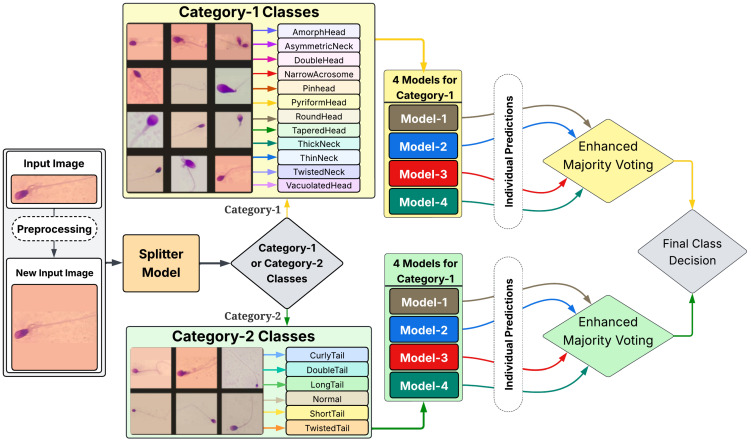
Overview of the two-stage classification pipeline. This enlarged diagram illustrates preprocessing, the splitter model, routing into Category 1 (head and neck abnormalities) versus Category 2 (normal and tail abnormalities), and the category-specific ensembles with their enhanced majority voting scheme for final class decisions.

The Hi-LabSpermMorpho dataset, comprising the BesLab, HistoPlus, and GBL sub-datasets categorized by staining methods, contains cropped images of individual sperm exhibiting diverse morphological abnormalities. Figure 1 now presents representative examples from both Category-1 classes and Category-2 classes for the BesLab dataset. Since sperm cells appear in various orientations and aspect ratios, the images have variable dimensions. To standardize the inputs, each sample is padded with the average background color to form a square shape before resizing.

To ensure an unbiased evaluation, the dataset was divided into training and validation sets using a stratified approach. Instead of applying a single transformation pipeline to the entire dataset, separate preprocessing pipelines were created for training and validation. The training data was augmented using strong transformations, whereas the validation data was only resized and normalized. This separation ensured that the validation accuracy reflected the model’s generalization ability without augmentation-induced artifacts.

### 2.2. Two-Stage Classification Pipeline

A distinctive two-stage classification pipeline is implemented, initially sorting images into two general morphological categories before applying ensemble classification. Figure 1 presents the two-stage classification pipeline with its application details delineated below.

#### 2.2.1. Stage 1: Splitter Model

The first stage of the classification pipeline consists of a binary classifier, referred to as the splitter model. This model determines whether an image belongs to the following:

- **Category 1:** Sperm abnormalities primarily related to the head and neck region. This includes 12 classes: *AmorfHead, AsymmetricNeck, DoubleHead, NarrowAcrosome, PinHead, PyriformHead, RounHead, TaperedHead, ThickNeck, ThinNeck, TwistedNeck,* and *VacuolatedHead*.
- **Category 2:** Normal sperm morphology and tail-related abnormalities. This includes 6 classes: *CurlyTail, DoubleTail, LongTail, Normal, ShortTail,* and *TwistedTail*.

The splitter model was trained using a subset of the dataset in which each image was assigned to either Category 1 or Category 2. The architecture was selected based on empirical results. Although standard ViT models underperformed in morphology classification, the architecture surprisingly outperformed other models in binary classification and was selected as the primary model for the splitter due to its superior performance in distinguishing between the two categories.

#### 2.2.2. Stage 2: Category-Specific Ensemble Classifiers

Once an image is classified into a category, it is processed by an ensemble of models specifically trained in the classes of that category. Each category consists of an ensemble of four independently trained deep neural networks. The ultimate prediction is determined by majority voting across these models. Four identical models are applied to Categories 1 and 2, resulting in eight models within the system, excluding the splitter model.

The architectures selected for each ensemble include the following:

- **ViT-Huge (CLIP Laion-2B):** A vision transformer pretrained on approximately 2 billion image–text pairs, achieving robust generalization capabilities without fine-tuning [28]. Due to its extensive pretraining, it captures diverse visual features well-suited to sperm image variability.
- **ViT-Large (CLIP OpenAI)**: A smaller ViT variant trained on the CLIP dataset by OpenAI [29], known for effective semantic understanding and robustness to image variability. Its moderate size makes it well-suited for fine-tuning on the dataset used in this study.
- **ViT-Large (ImageNet-21k)**: A purely supervised vision transformer pretrained on the extensive ImageNet-21k dataset (14 million images, 21,843 classes) [30]. This model demonstrated strong baseline accuracy for fine-grained sperm classification tasks.
- **DeepMind NFNet-F4**: A high-performance CNN architecture known for robust feature extraction without normalization layers, enabling stable training and excellent transfer learning capabilities [31]. Its strengths lie in capturing localized morphological details critical for identifying specific abnormalities.

Each model was fine-tuned independently on the sperm morphology dataset using transfer learning. By combining transformers and CNN architectures with distinct training regimes, the objective is to identify the model that best balances generalization and specialization for sperm morphology.

Regarding the ensemble learning, each individual model in the ensemble may capture different aspects of the data’s features or may make different errors [32,33]. By assembling heterogeneous models, the ensemble capitalizes on their complementary strengths. Effectively, the fused classifier can focus on a broader range of discriminative features than any single model while balancing out individual model weaknesses. Ensemble learning leverages model diversity to reduce prediction errors arising from intra-class variability and limited dataset sizes [32,33]. One model might be better at recognizing subtle elongated head shapes, while another identifies slight asymmetries effectively. Through majority voting, the ensemble capitalizes on these varied strengths, significantly mitigating individual model biases and ensuring stable, reliable classification performance.

### 2.3. Model Architectures and Training Strategy

Firstly, we evaluated several architectures (ViT, EfficientNetV2, ConvNeXt and NFNet); NFNet models, especially dm_nfnet_f4, consistently achieved the highest accuracy.

#### Training Details

- **Data Augmentation:** Augmentations (random flips, rotations, affine transforms, and random erasing) were applied only to the training set, increasing its effective size by 3.2 times. The validation set remained unchanged.
- **Multi-Phase Training Strategy:** A three-phase curriculum was employed for fine-tuning the pretrained model on the target dataset. This multi-phase training strategy—consisting of an initial warm-up, followed by head-only training, and finally gradual unfreezing of the backbone—is designed to stabilize training and maximize transfer learning performance. Similar phased fine-tuning approaches have been advocated in prior studies (in both general transfer learning and biomedical imaging contexts) to avoid catastrophic forgetting and to better leverage pretrained features [34,35]. All phases were executed sequentially as a single continuous training schedule, without resetting the optimizer or weights between phases. Each phase and its rationale are detailed below.
  1. **Phase 1: Warm-Up Full-Network Fine-Tuning (Stabilization):** All network layers were briefly fine-tuned (1–2 epochs) with a low learning rate, stabilizing pretrained weights and adjusting initial feature representations to the dataset.
  2. **Phase 2: Freeze Backbone and Train Classifier Head (Linear Probing):** The backbone was frozen, training only the classifier head. This allowed the model to rapidly learn decision boundaries specific to sperm morphology while preserving general pretrained features.
  3. **Phase 3—Gradual Unfreezing:** Layers of the backbone were gradually unfrozen from deepest to shallowest. Differential learning rates—higher for the classifier head and lower for backbone layers—facilitated controlled adaptation to the new task.
- **Training Protocol and Hyperparameters:** Training phases proceeded sequentially without optimizer resets. The AdamW optimizer with cosine annealing learning rate scheduling was used. Mixed-precision training reduced memory consumption, permitting larger batch sizes. Training continued up to 150 epochs with early stopping (patience of 12 epochs), selecting the checkpoint with the highest validation accuracy.

### 2.4. Evaluation Metrics and Ensemble Decision

Performance was assessed with the standard class-wise *precision* (${\mathrm{Pr}}_{k}$), *recall* (Rek), and *F*_1_-score ($F_{1,k}$) for each class k(k=1,…,K), defined as

(1) $$\begin{array}{cc} {\mathrm{Pr}}_{k} & =\frac{{\mathrm{TP}}_{k}}{{\mathrm{TP}}_{k}+{\mathrm{FP}}_{k}}, \end{array}$$

(2) Rek=TPkTPk+FNk,

(3) F1,k=2PrkRekPrk+Rek,

where ${\mathrm{TP}}_{k}$, ${\mathrm{FP}}_{k}$, and ${\mathrm{FN}}_{k}$ denote the true-positive, false-positive, and false-negative counts for class *k*, respectively. Because the dataset is highly imbalanced, the *macro-averaged* F_1_-score is reported, which assigns equal weight to every class,

(4) $$F_{1}^{\mathrm{macro}}=\frac{1}{K}\sum_{k=1}^{K}F_{1,k}.$$

**Ensemble Decision.** At inference time, the *Splitter Model* first routes each sample to either *Category 1* or *Category 2*. Within the selected category, an ensemble of *M* classifiers ${\left\{f_{m}\right\}}_{m=1}^{M}$ produces class labels ${\hat{y}}_{m}\in \left\{1,\ldots ,K_{\;c}\right\}$, where $K_{\;c}$ is the number of classes in that category. The final prediction is obtained via Two–Stage Ensemble Fusion Strategy.

### 2.5. Two–Stage Ensemble Fusion Strategy

Let C={1,…,K} be the set of class labels and let M={1,…,M} denote the ensemble of M=4 base models. For a test sample x, each model m∈M outputs a posterior distribution $p_{m}\left(x\right)=\left[p_{m,1},\ldots ,p_{m,K}\right]$, with $\sum_{k=1}^{K}p_{m,k}=1.$

(5) $$\begin{array}{cccc} t_{m} & =\arg\max_{k\in C}p_{m,k},\; & \; & (\mathrm{top}-1\;\mathrm{vote}), \end{array}$$

(6) $$\begin{array}{cccc} s_{m} & =\arg\max_{k\in C\setminus \{t_{m}\}}p_{m,k},\; & \; & (\mathrm{top}-2\;\mathrm{vote}). \end{array}$$

**Stage 1—Majority over top-1 votes.** For every class *k*, count

(7) $$c_{k}^{\left(1\right)}\;=\;\sum_{m=1}^{M}I\;\left(t_{m}=k\right),$$

where I(·) is the indicator function. Let $k_{1}^{*}=\arg\max_{k}c_{k}^{\left(1\right)}$ and denote the maximal vote count by $C_{\max}^{\left(1\right)}=c_{k_{1}^{*}}^{\left(1\right)}.$ If $k_{1}^{*}$ is *unique*, i.e., $\left|\{k:c_{k}^{\left(1\right)}=C_{\max}^{\left(1\right)}\}\right|=1,$ it is assigned as the prediction

(8) $$\hat{y}=k_{1}^{*},$$

and terminated; otherwise the process proceeds to Stage 2.

**Stage 2—Augmented vote aggregation.** Construct an augmented vote set $V_{m}=\left\{t_{m},\;s_{m}\right\}$ and compute the combined counts

(9) $$c_{k}^{\left(2\right)}\;=\;\sum_{m=1}^{M}\;\left[\;I\;\left(t_{m}=k\right)\;+\;I\;\left(s_{m}=k\right)\right].$$

Let $k_{2}^{*}=\arg\max_{k}c_{k}^{\left(2\right)}$ with maximal count $C_{\max}^{\left(2\right)}=c_{k_{2}^{*}}^{\left(2\right)}.$ If $k_{2}^{*}$ is *unique*, then

(10) $$\hat{y}=k_{2}^{*}.$$

If a tie persists, the prediction of the *reference model* $m^{†}$—the single model exhibiting the highest validation accuracy—is used as a fallback:

(11) $$\hat{y}=t_{m^{†}}.$$

**Figure 2:**
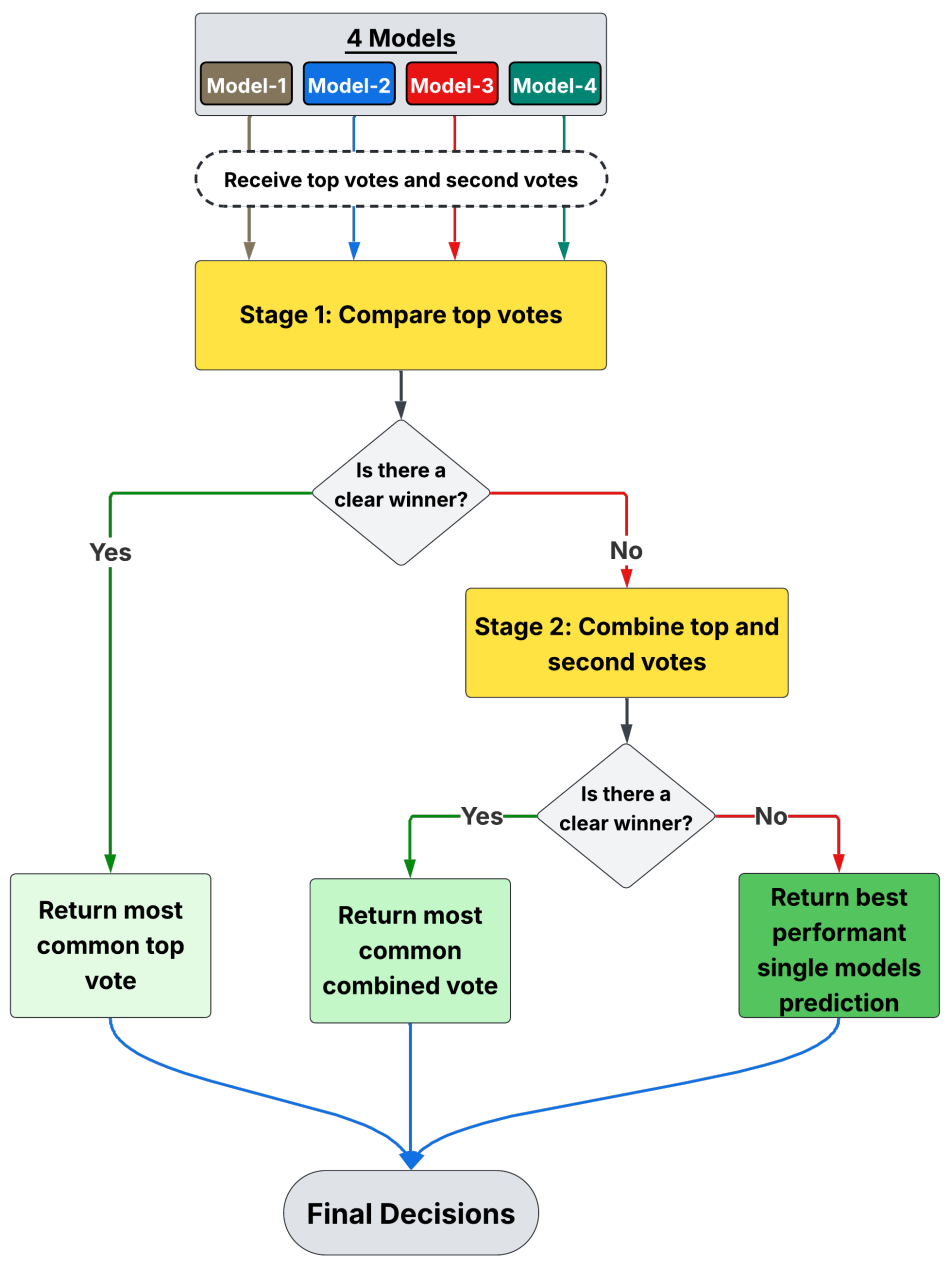
Majority voting strategy for ensemble models. Each model in the category ensemble predicts a class, and the final class is determined by the most frequent prediction.

Equation (7) enforces a strict majority over the most confident votes; when no single class dominates, the augmented rule (9) leverages secondary preferences to disambiguate borderline cases. This two-stage scheme (Figure 2) maintains high precision when the models agree, while Equation (11) guarantees a deterministic outcome under residual ties, ensuring robustness and reproducibility.

This ensemble fusion mechanism is inspired by voting-based ensemble strategies in deep learning [36], but features a novel two-stage design leveraging both top-1 and top-2 model confidences.

## 3. Experimental Results

### 3.1. Splitter Model and Split Category Analysis

**Figure 3:**
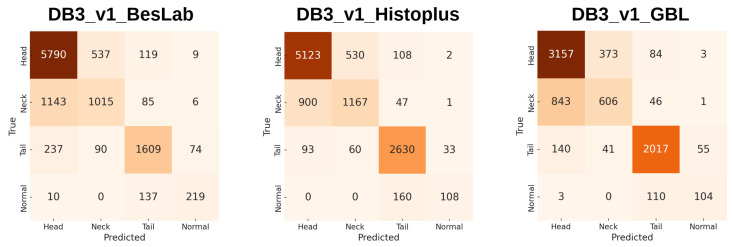
ViT-Large (ImageNet-21k) performance with a four-category division on a sub-dataset each belonging to one of the three staining techniques, showing accuracies around 80%.

**Figure 4:**
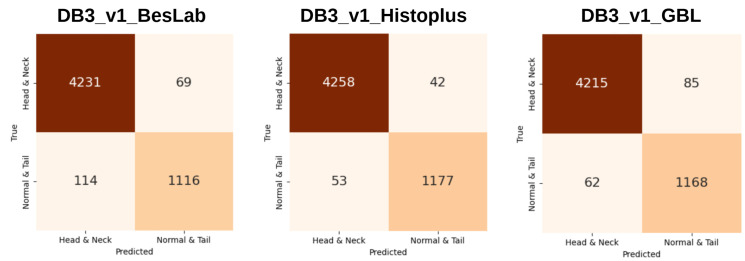
ViT-Large (ImageNet-21k) performance with a two-category division on a sub-dataset each belonging to one of the three staining techniques, showing accuracies around 97%.

In early experiments, multiple approaches were explored to split the dataset, including divisions into four or three categories and merging groups with high false-positive rates. After several trials, a consistent pattern was observed: models naturally tended to confuse head and neck abnormalities with each other, while tail abnormalities and normal sperm were often misclassified together. Figure 3 and Figure 4 illustrate the performance (average of five folds) of models on a four-category split, where accuracy remained around 80%, and a two-category split, where accuracies for all staining methods improved to the 97–98% range. These findings strongly support the viability of a two-category solution, which was subsequently adopted as the final approach.

Despite being mostly the worst performer as a sperm classifier, ViT-Large (ImageNet-21k) became the best splitter model, as shown in Table 2 for the BesLab stained samples. This indicates that a model with large-scale pretraining and a global attention mechanism can capture the salient features that differentiate the two categories especially well. In statistical terms, the first-stage splitter tends to lower the Bayes error—the irreducible error that arises when classes overlap—because it carves the feature space into two coarse groups (head/neck vs. tail/normal) whose internal classes are far less confusable than the original 18-way mix. By routing each sample to the “right neighborhood” before fine-grained classification, the downstream models face a simpler, cleaner decision boundary, so they can devote their capacity to learning the subtle differences that remain and achieve markedly higher accuracy.

After the determination of ViT-Large (ImageNet-21k) as the best splitter network by the experimentation of various hyper-parameters and model types, the same architecture was also applied to Histoplus and GBL stained images with the aim of first-level data splitting. The obtained results of splitting for each dataset are shown in Table 3.

### 3.2. Experimental Results of Individual Models Applied After Splitting

Following the dataset splitting, model performance was evaluated separately on Category 1 (head/neck abnormalities) and Category 2 (tail abnormalities and normal sperm). As summarized in Table 4, classification for Category 1 proved significantly more challenging, reflecting greater complexity and inter-class visual similarity. While the top-performing model (DeepMind NFNet-F4) achieved Category 1 accuracies of approximately 64–66%, models easily reached accuracies exceeding 80% for Category 2. This performance gap aligns with the use of separate specialized architectures for each morphological category.

Another observation was the distinct performance patterns of individual architectures between categories. DeepMind NFNet-F4 demonstrated a clear strength in Category 1 (likely due to superior texture analysis capabilities) while transformer models performed relatively better for Category 2. These results justify the use of different model architectures tuned to each subset of classes to use the benefits of architectural strengths optimally.

### 3.3. Choosing the Right Models for Ensemble Methods

A common mistake in ensemble learning is selecting only the highest-accuracy models and expecting an optimal result. However, experimental findings revealed that model diversity is essential—ensemble models must complement each other to achieve the best results. Training experiments showed that models generally fell into two distinct performance types: (1) Models with high recall for Amorf Head, but weak recall on smaller classes. These models achieve high accuracy primarily by correctly classifying the dominant class. (2) Models that struggle with Amorf Head but perform well across all classes achieve balanced accuracy.

In a typical ensemble, an odd number of models is preferred to prevent ties. However, due to the strong dominance of the two model types described above, an odd-numbered ensemble led to one type consistently overpowering the other, rendering the ensemble ineffective. To address this issue, an even number of models was purposely used, selecting two from each performance category to create a balanced ensemble.

#### 3.3.1. Handling Draws in Ensemble Predictions

With an even number of models, ties inevitably occurred. Several approaches were tested to resolve them, including custom rule-based tie-breaking methods (e.g., prioritizing Amorf Head if the tie was between two classes). However, such methods risked overfitting to the validation set rather than ensuring generalization.

Ultimately, a multi-stage ensemble voting strategy was adopted, which resolved ties in a structured manner.

**Steps of Multi-Stage Ensemble Voting**

- **Step 1: Primary Voting Round**
  Each model votes for its top predicted class.
- **Step 2: Secondary Voting Round**
  If no class wins an outright majority, the second-choice votes from each model are included in the tally.
- **Step 3: Fallback Decision**
  If a tie persists after both voting rounds, the final decision defers to the model with the highest validation accuracy (NFNet-F4 in this case). This step is rarely needed but ensures a definitive prediction.

Multiple variations of this strategy were explored, such as introducing a third voting round and combining first- and second-choice votes in the initial step. However, the proposed two-stage voting system with a fallback decision mechanism achieved the best balance of accuracy and robustness. A detailed mathematical formulation of this novel fusion technique is provided in Section 2.4.

#### 3.3.2. An Ablation Study to Find the Optimum Architecture

The proposed split-and-ensemble method consistently achieved the highest accuracy (68–71%). Table 5 summarizes the final accuracy achieved in each scenario across the three datasets: DB3_v1_BesLab, DB3_v1_Histoplus, and DB3_v1_GBL. Ensemble learning alone already outperformed single-model baselines, yet the hierarchical splitting further improved performance by addressing both inter-category and intra-category classification errors. In contrast, the single-model baseline achieved significantly lower accuracy (approximately 3–5 percentage points lower), highlighting the difficulty of handling all 18 classes simultaneously without the proposed strategies.

Overall, the split-and-ensemble framework provided clear improvements without requiring additional data or manual feature engineering, validating the efficiency of the proposed method for automated sperm morphology classification.

Several insights can be drawn from these results. First, the benefit of model ensembling is clear: in both split and non-split training methods, the ensemble consistently outperforms a single model. By combining predictions from multiple models, the system can correctly mitigate model errors and reduce variance, yielding a more robust prediction.

Second, and most importantly, the combination of splitting + ensembling consistently provides the largest gain across all datasets. This suggests that the two techniques address different aspects of the problem in a complementary way. The splitter tackles inter-category confusion by routing images to the appropriate expert models, effectively reducing the 18-class problem into two smaller sub-problems with less overlap. This alone improves accuracy (as seen clearly by comparing the Splitter + Best Model scenario vs. the single-model baseline). Indeed, a hierarchical divide-and-conquer strategy tends to mitigate confusions between very dissimilar classes. For instance, without splitting, a normal-tail sperm might occasionally be confused with a tapered-head sperm by a single classifier, whereas in the split approach such a mix-up is nearly impossible because the tail image would be classified only among tail classes. On top of this, ensembling addresses intra-category ambiguities by averaging out the idiosyncrasies of different models. The full system (Splitter + Ensemble) thus enjoys both reduced category-level mistakes and reduced model-specific errors, culminating in the highest accuracy. In Category 1, where classes are numerous and harder to distinguish, the ensemble contributes a particularly notable improvement over the single model (bridging many of the gaps noted in Table 4). In Category 2, the gain from ensembling, while still present, is smaller simply because a single model can already handle the easier tail classification fairly well.

Finally, the baseline single-model approach consistently had the lowest accuracy among the four configurations across all datasets, underscoring the difficulty of the task when tackled in one monolithic step. The gap between the baseline and the proposed method is substantial (approximately 3–5 percentage points in accuracy), a significant margin in a task of this complexity. This reinforces the merit of the proposed approach. Overall, the consistent ranking of the four configurations (Splitter + Ensemble > Splitter + Best > Ensemble without split > Single model) validates both hierarchical splitting and ensemble voting as effective strategies for improving sperm morphology classification. These improvements are achieved without introducing additional data or manual features—they stem purely from smarter utilization of model architectures and their combination. The outcome aligns well with general ensemble theory and hierarchical learning principles, advocating that dividing a problem and aggregating multiple classifiers yields better generalization than using a single, flat classifier.

### 3.4. Confusion Matrix-Based Results

**Figure 5:**
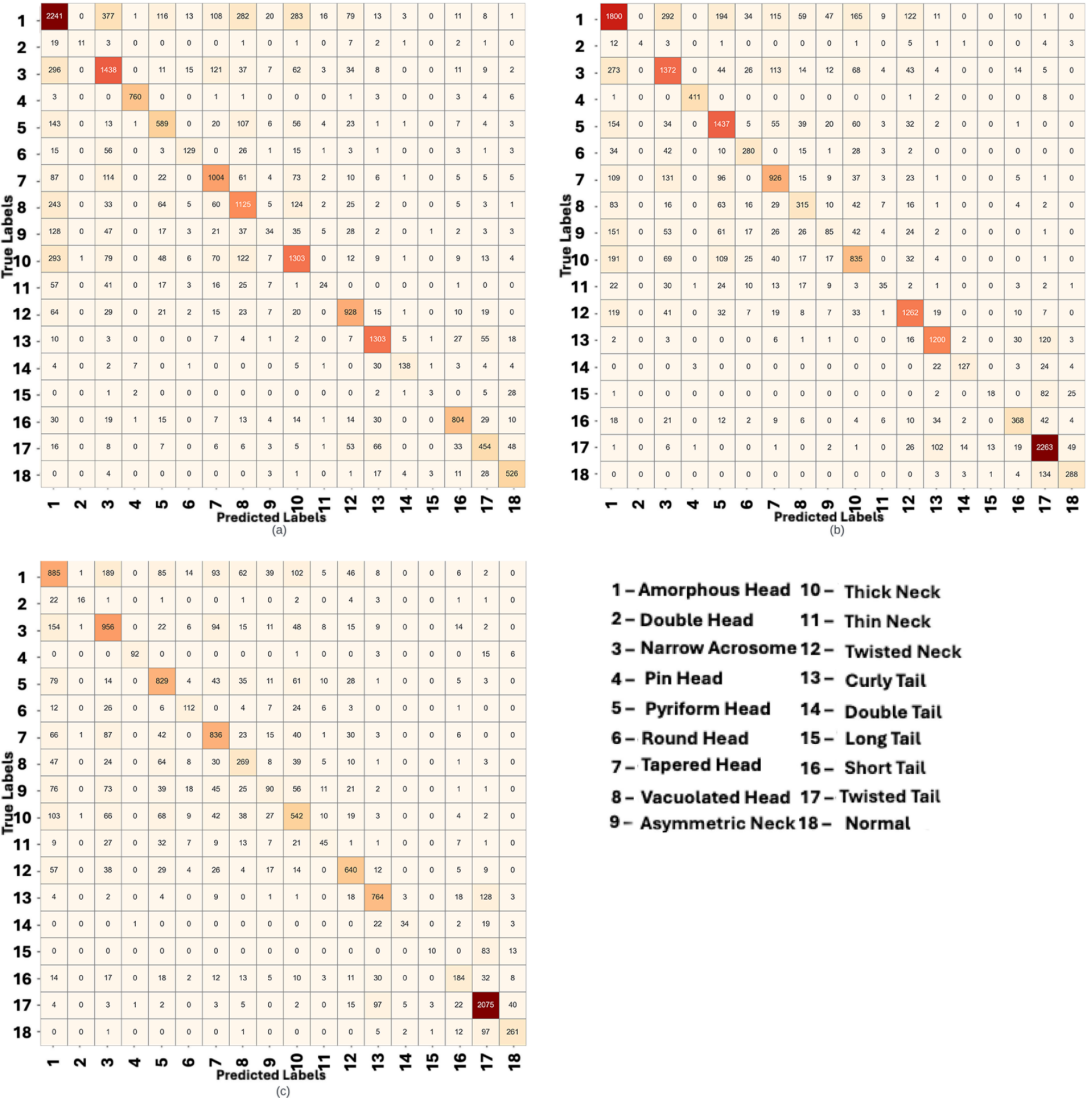
Five-fold cross-validation cumulative results of the final classification system (Splitter + Ensemble) presented as a confusion matrix. Each cell shows the number of instances of a true class (rows) that were predicted as a given class (columns), with darker colors indicating higher counts (the diagonal cells represent correct predictions for each class). The confusion matrix highlights which classes are most often mistaken for each other. Models was trained on three datasets: DB3_v1_BesLab (**a**), DB3_v1_Histoplus (**b**), and DB3_v1_GBL (**c**). Each confusion matrix shows result for different corresponding datasets.

The confusion matrix in Figure 5 illustrates key strengths of the proposed model, including highly accurate classification of classes such as PinHead, CurlyTail, and ShortTail. Nonetheless, notable misclassifications occur among visually similar categories, for example, between ThickNeck and VacuolatedHead, and the LongTail class remains particularly challenging, with a high rate of incorrect predictions. Importantly, most misclassifications are contained within the same broad category (head/neck vs. tail/normal), demonstrating the effectiveness of the initial splitter in separating major morphological groups.

Table 6 provides detailed per-class metrics, confirming the trends observed in the confusion matrix. Overall accuracies reached 69.43% for DB3_v1_BesLab, 71.34% for DB3_v1_Histoplus, and 68.41% for DB3_v1_GBL, underscoring the model’s consistent performance across datasets. The detailed analysis presented here focuses primarily on DB3_v1_BesLab, which is representative of general model behavior. Looking at individual classes, the PinHead class stands out with an F1-score of 0.978—extremely high performance—stemming from both precision 0.984 and recall 0.972. CurlyTail and ShortTail also show strong F1-scores (approximately 0.88 and 0.83, respectively), indicating these tail deformities are reliably recognized. The model also performs well on normal sperm, with an F1 around 0.83 and recall 0.88, which is important since normal is a relatively smaller class here (only 598 samples in the test set), yet achieving high recall means the system has a low false-negative rate for normal sperm. These high-performing classes tend to be ones with distinctive morphology and/or plentiful training examples. For instance, TwistedNeck has an F1 of ∼0.78 with recall 0.804, benefitting from a decent sample size and a clear curved-neck appearance that the model can latch onto.

On the other end, the model fails to detect most LongTail instances (recall 7%), often misclassifying them into a wide range of other classes—such as Normal, ShortTail, or CurlyTail—rather than consistently confusing them with a specific category. This scattered misclassification pattern, visible as a dispersed row in the confusion matrix (Figure 5), reflects the model’s uncertainty and difficulty in learning distinctive features for the LongTail class. ThinNeck and AsymmetricNeck are two other classes with F1 below 0.20. AsymmetricNeck (precision 0.31, recall 0.09) indicates that out of 366 asymmetric neck cases, the model identified fewer than 10% correctly—an extremely low recall. Many of those were likely labeled as normal or other head defects (since an asymmetric neck might not be dramatically different in shape). ThinNeck likewise had recall 0.125 (12.5%), suggesting the decision boundary for thin vs. normal neck is poorly learned. DoubleHead is an interesting case with precision 0.916 but recall only 0.229: the model almost never wrongly predicts double-head (hence high precision), but it also misses the majority of true double-head instances. This is mostly caused by having a really low sample size (only 48 samples) while having very distinctive features; double head is, even for the human eye, the easiest class to identify. Such behavior is common in class-imbalanced scenarios where the model essentially ignores the minority class to optimize overall accuracy. In fact, the macro-averaged metrics (precision 0.686, recall 0.594) are much lower than the weighted averages, reflecting that the model performs substantially worse on the smaller classes compared to the dominant ones.

From a practical standpoint, these per-class results highlight both the strengths and the remaining challenges of the split-and-ensemble approach. The high precision and recall for several defect types demonstrate that the system can be trusted to reliably identify those anomalies (e.g., PinHead or CurlyTail are rarely misidentified). The errors are largely concentrated in classes that even human experts might confuse or that had very few training examples. Misclassifications such as thick vs. twisted neck or failing to catch a subtle asymmetric neck point to intrinsic difficulties in the visual differentiation of those features. The presence of such errors is not surprising—class imbalance and subtle morphological differences are well-known hurdles in medical image classification, often causing minority classes to be overshadowed by majority classes. Nonetheless, the fact that the system achieves nearly 70% overall accuracy across 18 classes (including many challenging ones) is encouraging. It suggests that the combination of splitting and ensembling successfully handled a large portion of the variability.

In summary, the quantitative findings summarized in Table 4, Table 5 and Table 6 and visualized in Figure 5 confirm both the strengths and remaining challenges of the proposed split-and-ensemble strategy. Table 4 shows that Category-2 classes (tail defects and normal sperm) are generally easier to classify, reaching accuracies above 80%, whereas Category-1 classes (head and neck defects) remain more difficult, reflecting greater morphological variability. The ablation results in Table 5 further highlight that hierarchical splitting combined with ensemble learning achieves the highest overall accuracy across all staining protocols, outperforming single-model and unsplit configurations by 3–5 percentage points. The confusion matrix in Figure 5 illustrates that misclassifications remain within the same major category, validating the effectiveness of the splitter. Classes with distinctive morphology and ample training data, such as PinHead, CurlyTail, and ShortTail, achieve high F1-scores (≥0.83), whereas rare or subtle classes, including LongTail and ThinNeck, show lower recall due to class imbalance and feature similarity. Table 6 provides a detailed per-class performance summary, underlining that precision remains high for several minority classes (e.g., DoubleHead) despite low recall, suggesting that increasing the representation of rare anomalies could further boost performance. Together, these results emphasize that the two-stage pipeline not only enhances classification reliability but also offers interpretable performance patterns aligned with clinical observations of sperm morphology variability.

## 4. Discussion

The findings of this study show that using a two-stage classification approach improves the accuracy of sperm morphology classification accuracy, achieving overall accuracies of 69.43%, 71.34%, and 68.41% across the three staining-specific datasets (DB3_v1_BesLab, DB3_v1_Histoplus, and DB3_v1_GBL, respectively). Starting with categorizing images into two broad groups, head and neck abnormalities versus tail abnormalities and normal samples, the model substantially reduces confusion among visually similar classes. Our results align with previous research indicating that head morphology significantly influences sperm classification [2].

One key observation was that normal sperm and tail abnormalities are more frequently confused with each other than with head/neck abnormalities, highlighting the dominant role of head morphology in current model predictions. This suggests that future work might benefit from models or preprocessing steps that explicitly focus on specific sperm regions like cropping only head or tail parts to separate different parts of the sperm.

In this study, our main focus was the head and neck abnormality category, as it makes up 78.44% of the dataset and showed lower classification performance compared to the tail and normal category. Ensemble learning significantly improved the results in this category, outperforming individual models. Unlike typical ensembles that use an odd number of models to avoid ties, we designed an even-numbered ensemble to balance two distinct classification patterns observed in our experiments. To resolve ties in ensemble predictions, we introduced a multi-stage ensemble voting strategy. Instead of a simple majority vote, we collected both primary and secondary predictions from each model, allowing for a more flexible decision-making process. This approach proved more effective than fixed rule-based methods, reducing classification errors while maintaining generalization.

Notably, the overall accuracy of the proposed model must be interpreted in context. Prior studies that focus on a narrower set of sperm categories have reported higher accuracies. For instance, classifying only sperm head-shape abnormalities can achieve over 90% accuracy on dedicated datasets. The HuSHeM benchmark dataset, for example, has seen models like VGG16 reach 94% accuracy on just four head-shape classes.In contrast, our approach tackles all major sperm abnormalities across 18 categories, representing a much more difficult problem. The fact that the proposed two-stage system can handle this complexity with approximately 70% accuracy across three different staining protocols is a promising result, suggesting that deep learning can be scaled up to comprehensive sperm morphology analysis, beyond the limited scope of earlier works.

### 4.1. Comparison with Baseline Results on the Hi-LabSpermMorpho Dataset

The original Hi-LabSpermMorpho dataset study provided a strong baseline, evaluating 35 deep learning models for single-stage sperm morphology classification. Their best results were achieved with EfficientNet-V2 models, which reached 65.05% accuracy on the BesLab dataset, 67.42% on Histoplus, and 63.58% on GBL [2].

In comparison, our two-stage split-and-ensemble approach achieves higher accuracy on all three datasets—69.43% on BesLab, 71.34% on Histoplus, and 68.41% on GBL—showing absolute improvements of 4–5%. This gain is mainly due to the proposed hierarchical framework, which first divides the problem into two main categories and then applies an ensemble of ViT and NFNet models for detailed classification within each group. These results confirm existing findings supporting ensemble learning to achieve more robust predictions in sperm morphology classification.

### 4.2. Effect of Splitting and Ensemble Methods on Accuracy

The proposed hierarchical split-and-ensemble strategy significantly improved accuracy, outperforming both individual models and non-split ensembles. The first-stage “splitter,” a ViT-Large (ImageNet-21k), showed almost perfect category-level discrimination (97–98% accuracy). This effectively simplified downstream classification tasks, similar to recent studies highlighting transformers’ global attention mechanism advantage for medical image tasks [37].

In the second stage, category-specific ensembles consistently achieved higher accuracy compared to single models. This was mostly visible in head and neck classes, increasing accuracy from around 59–66% to 63–68%. Even the simpler tail/normal category saw accuracy improvements (from 77–82% to 81–85%), underscoring ensemble methods’ potential to correct complementary model errors. These results confirm existing findings supporting ensemble learning to achieve more robust predictions in sperm morphology classification [38].

In our study, when one model struggles with a borderline example (e.g., a borderline thin vs. normal neck), another model in the ensemble may correctly identify it, and the voting scheme can then select the right label. This highlights an advantage of ensembles in medical image classification – they reduce the variance of predictions and can capture complementary features learned by different architectures. The trade-off, of course, can increase computational complexity, as noted by other authors who caution that very large ensembles may become impractical for real-time clinical use [39]. In our implementation, we mitigated this by using a tie-break voting strategy (Stage1 and Stage2 voting) to keep the ensemble small and efficient, while still reaping most of the accuracy benefits.

### 4.3. Future Work and Limitations

Although the split-and-ensemble strategy achieved improved classification accuracy, several important limitations remain, highlighting directions for future work. Class imbalance is a major challenge: some abnormality classes in the dataset contained only a few dozen samples (e.g., LongTail or DoubleHead), restricting the model’s ability to learn their unique features. This limitation is evident in the low recall rates and frequent misclassification of these rare classes. Addressing data imbalance will therefore be critical in future research. Potential approaches include advanced data augmentation techniques—such as scaling, rotation, and synthetic distortion of sperm images—to increase the diversity and representation of under-represented categories. More ambitiously, generation of realistic sperm images for under-represented classes using approaches like generative adversarial networks (GANs) could provide additional training data. Recent research has already shown the promise of GAN-based augmentation for sperm morphology—Jabbari et al. combined a conditional GAN with a Capsule Network to synthesize minority-class sperm head images, markedly improving classification of rare defects [40]. Incorporating such synthetic data generation or advanced augmentation into our pipeline could bolster the recognition of anomalies like ThinNeck or LongTail, which currently have high misclassification rates.

Another limitation lies in the reliance on the initial category classifier. While our splitter was 97% accurate, the 3% of samples that it misroutes will almost certainly be misclassified in the second stage (since they go to the wrong specialist ensemble). In practice, this means a small fraction of sperm (especially those near the decision boundary of two categories) might be systematically handled by an inappropriate classifier. One possible improvement is to make the system more uncertainty-aware. For example, if the splitter is unsure between two categories for a given image (i.e., the prediction confidence is low or two category probabilities are close), the system could forward the image to both corresponding specialist models and then reconcile the outputs. This would increase computation slightly but could rescue borderline cases. Another approach is to train a unified second-stage model that can correct splitter errors—for instance, by including an “other” class in each specialist that triggers when the input does not match its category, prompting re-routing. These are architectural refinements that could make the pipeline more fault-tolerant.

Furthermore, morphological overlap and labeling ambiguities pose challenges that our current model does not explicitly address. In some cases, the distinction between classes is not crisp: for example, differentiating a mildly TaperedHead from a ThickNeck abnormality might depend on subjective judgment of where the “head” ends and “neck” begins. It is possible that some sperm flagged as errors by our model are actually borderline cases even for human experts. Future work could explore incorporating morphometric measurements or domain constraints to aid in decision-making for such cases. Another direction is to integrate segmentation of sperm parts into the pipeline. Our model classifies based on whole-sperm images (or crops thereof), but providing the network with explicit segmented regions (head, midpiece, tail) could improve focus on shape details. Recent studies have demonstrated that combining segmentation with classification—e.g., using mask-guided feature extraction—can enhance sperm morphology recognition. For instance, Sapkota et al. introduced a mask-guided feature fusion that uses sperm head segmentation to guide the classification network, achieving record-high accuracy [41]. Adopting a similar idea, we could first isolate the sperm’s head and tail with a segmentation model, then apply the split classification. This might reduce confusion between, say, a ThinNeck vs. TaperedHead by ensuring the model only looks at the relevant anatomical region when making that fine distinction.

Finally, while our method integrates both transformer-based (ViT) and convolutional (NFNet) architectures, further improvements could be made by developing hybrid models that combine both approaches. Transformers are really good at capturing long-range dependencies, while CNNs are better at local feature extraction. Future models built for sperm morphology analysis could integrate both mechanisms for improved accuracy.

From a clinical perspective, a key limitation is that our system was developed and evaluated on a specific dataset of stained sperm microscope images. Generalization to other imaging conditions (e.g., unstained live sperm imaging, different microscopes or labs) is not guaranteed. The model may need retraining or fine-tuning on data from each new setting to maintain accuracy. Additionally, the current deep models (especially the ViT-Large) are computationally intensive, which could hinder real-time deployment in routine laboratory workflows. However, as hardware accelerators improve and techniques like model compression or knowledge distillation are applied, we anticipate these models can be optimized for speed and even run on-premise in clinics. Validation in a real clinical workflow, perhaps as a decision-support tool alongside embryologists, would be an important next step. We plan to collaborate with clinical laboratories to test the system’s performance on prospective samples and to gather user feedback.

## 5. Conclusions

In this work, we introduced a novel two-stage split-and-ensemble deep learning framework for comprehensive classification of human sperm morphology across three staining-specific datasets (DB3_v1_BesLab, DB3_v1_Histoplus, and DB3_v1_GBL). The method employs a coarse-to-fine strategy: a transformer-based model (ViT-Large) first classifies each sperm image into a broad defect category with high accuracy (≈97%), after which an ensemble of specialized CNN/transformer classifiers identifies the specific morphological abnormality. This design effectively manages the large variability and class imbalance inherent in sperm morphology datasets, outperforming conventional single-model classifiers and achieving top overall accuracies of 69.43%, 71.34%, and 68.41% (macro-F1 ≈ 0.59–0.62) across the full 18-class problem.

Notably, the proposed framework excels in recognizing frequently occurring and visually distinctive defects such as pin-headed sperm, achieving near-perfect accuracy while also demonstrating strong performance in detecting a diverse range of head, midpiece, and tail abnormalities within a single unified system. This breadth of classification extends beyond the focus of most prior works, which typically target only head shape defects [18,42].

The quantitative evaluation summarized in Table 4, Table 5 and Table 6 and visualized in Figure 5 reinforces the effectiveness of the proposed two-stage split-and-ensemble framework. Across all three staining protocols, the method consistently achieved 3–5 percentage point accuracy improvements over the strongest single-model baseline, with particularly notable gains in the challenging head and neck abnormality category. The confusion matrix highlights that most misclassifications remain within the same broad category, validating the splitter’s role in reducing inter-class confusion, while Table 6 demonstrates strong precision for distinctive abnormalities and pinpoints areas for improvement, such as rare or visually subtle defects (e.g., LongTail and ThinNeck). These results confirm that the proposed strategy not only improves overall classification accuracy but also provides interpretable performance trends aligned with clinical observations, supporting its potential integration into future CASA systems.

In conclusion, the two-stage split-and-ensemble approach offers a promising and scalable path toward high-accuracy sperm morphology classification under multiple staining protocols. While class imbalance and cross-dataset generalizability remain challenges, the demonstrated improvements highlight the potential of ensemble deep learning models in medical imaging. With further optimization and clinical validation, the method could be integrated into next-generation CASA systems, enhancing diagnostic reliability and supporting improved reproductive health outcomes.

## Figures and Tables

**Table 1:** Class distribution of the Hi-LabSpermMorpho dataset: (a) BesLab, (b) Histoplus, (c) GBL [2].

	BesLab	Histoplus	GBL
Amorf Head	3572	2861	1537
Tapered Head	1399	1356	1150
Round Head	257	415	201
Pyriform Head	979	1843	1123
Pin Head	782	423	118
Vacuolated Head	1697	604	509
Double Head	48	35	52
Narrow Acrosome	2055	1992	1355
Twisted Neck	1154	1566	855
Thick Neck	1978	1340	934
Thin Neck	192	173	180
Asymmetrical Neck	366	492	458
Long Tail	42	128	106
Twisted Tail	706	2502	2277
Curly Tail	1445	1385	955
Double Tail	200	183	81
Short Tail	991	538	359
Normal	599	433	381
Total	18,462	18,269	12,631

**Table 2:** Accuracy of candidate splitter models for two categories applied to BesLab stained samples. Binary accuracy (%) indicates the percentage of images correctly split into head/neck and tail/normal categories; the highest accuracy model was selected as the splitter.

Model	Binary Accuracy (%)
ViT-Huge (CLIP, LAION-2B)	93.0
ViT-Large (CLIP, OpenAI)	94.0
ViT-Large (ImageNet-21k)	97.3
DeepMind NFNet-F4	96.2

**Table 3:** Accuracy of best splitter model (ViT-Large (ImageNet-21k)) across three datasets. Binary accuracy (%) indicates the percentage of images correctly split into head/neck and tail/normal categories for each staining method.

Model	Binary Accuracy (%)
DB3_v1_BesLab	97.3
DB3_v1_Histoplus	98.3
DB3_v1_GBL	97.5

**Table 4:** Per-category classification accuracy for each baseline model and the proposed ensemble. Category 1 includes head–neck abnormalities (12 classes), whereas Category 2 includes normal sperm and tail-related defects (6 classes).

Model	DB3_v1_BesLab		DB3_v1_Histoplus		DB3_v1_GBL	
	**Cat-1**	**Cat-2**	**Cat-1**	**Cat-2**	**Cat-1**	**Cat-2**
**Ensemble Method**	**67.92%**	**83.91%**	**68.01%**	**85.10%**	**62.81%**	**85.09%**
DeepMind NFNet-F4	65.90%	78.21%	65.95%	82.34%	62.00%	82.28%
ViT-Huge (CLIP Laion-2B)	60.40%	79.97%	62.67%	84.09%	58.11%	83.02%
ViT-Large (CLIP OpenAI)	60.37%	82.29%	63.13%	83.65%	57.47%	82.70%
ViT-Large (ImageNet-21k)	59.91%	78.34%	64.72%	82.10%	60.25%	81.80%

Note: Bold values indicate the best performance in each column.

**Table 5:** Overall accuracy (%) of four configuration strategies across the three datasets. The configurations compared are (1) Splitter Model + Ensemble, (2) Splitter Model + Best Model per Category, (3) Ensemble Without Splitting, and (4) Single Best Performer Model (NFNet-F4).

Configuration	DB3_v1_BesLab	DB3_v1_Histoplus	DB3_v1_GBL
Splitter Model + Ensemble	**69.43**	**71.34**	**68.41**
Splitter Model + Best Model per Category	67.55	69.61	67.21
Ensemble Without Splitting	67.49	69.37	67.09
Single Best Performer Model (NFNet-F4)	66.20	68.35	66.39

Note: Bold values indicate the best performance in each column.

**Table 6:** Classification report showing precision, recall, and F1-score for each of the 18 classes in the final model across three datasets (DB3_v1_BesLab, DB3_v1_Histoplus, and DB3_v1_GBL). Support (number of test samples per class) is listed to the right of each dataset. The overall accuracy, as well as macro-averaged and weight-averaged metrics, are provided at the bottom for each dataset.

Class	DB3_v1_BesLab				DB3_v1_Histoplus				DB3_v1_GBL			
	**P(%)**	**R(%)**	**F1(%)**	**Sup.**	**P(%)**	**R(%)**	**F1(%)**	**Sup.**	**P(%)**	**R(%)**	**F1(%)**	**Sup.**
AmorfHead	61.41	62.74	62.07	3572	60.59	62.96	61.75	2859	57.77	57.58	57.67	1537
AsymmetricNeck	31.19	9.29	14.32	366	38.64	17.28	23.88	492	37.82	19.65	25.86	458
DoubleHead	91.67	22.92	36.67	48	100.00	11.43	20.51	35	80.00	30.77	44.44	52
NarrowAcrosome	63.43	70.01	66.56	2054	64.93	68.88	66.85	1992	62.73	70.55	66.41	1355
PinHead	98.45	97.19	97.81	782	98.80	97.16	97.97	423	97.87	78.63	87.20	117
PyriformHead	63.33	60.22	61.74	978	68.99	78.01	73.22	1842	66.80	73.82	70.14	1123
RoundHead	72.88	50.19	59.45	257	66.35	67.47	66.91	415	60.87	55.72	58.18	201
TaperedHead	68.96	71.77	70.33	1399	68.49	68.29	68.39	1356	67.31	72.70	69.90	1150
ThickNeck	65.15	65.91	65.53	1977	63.31	62.31	62.81	1340	56.28	58.03	57.14	934
ThinNeck	40.00	12.50	19.05	192	46.67	20.23	28.23	173	43.27	25.00	31.69	180
TwistedNeck	75.76	80.42	78.02	1154	78.09	80.64	79.35	1565	74.33	74.85	74.59	855
VacuolatedHead	60.16	66.29	63.08	1697	59.21	52.15	55.46	604	52.95	52.85	52.90	509
CurlyTail	86.29	90.30	88.25	1443	85.05	86.71	85.87	1384	79.25	80.00	79.62	955
DoubleTail	88.46	69.00	77.53	200	85.23	69.40	76.51	183	77.27	41.98	54.40	81
LongTail	33.33	7.14	11.76	42	56.25	14.06	22.50	128	71.43	9.43	16.67	106
Normal	79.46	87.96	83.49	598	76.39	66.51	71.11	433	78.14	68.68	73.11	380
ShortTail	84.90	81.13	82.97	991	78.13	68.40	72.94	538	63.67	51.25	56.79	359
TwistedTail	70.39	64.31	67.21	706	83.91	90.59	87.12	2498	83.91	91.13	87.37	2277
Macro Avg	68.62	59.40	61.43	18,456	71.06	60.14	62.30	18,260	67.32	56.26	59.12	12,629
Weighted Avg	68.84	69.43	68.77	18,456	70.67	71.34	70.60	18,260	67.73	68.41	67.54	12,629
Overall Accuracy	**69.43**				**71.34**				**68.41**			

Note: Bold values indicate the overall accuracy for each dataset.

## Data Availability

The dataset utilized in this work, namely Hi-LabSpermMorpho, can be found in online repositories. Repository names and accession numbers: https://github.com/Yildiz-Hi-Lab/Hi-LabSpermMorpho accessed on 2 February 2025.
